# Cross species activity of TERT human telomerase component

**DOI:** 10.18632/aging.206372

**Published:** 2026-04-13

**Authors:** Sánchez-Vázquez Raúl, Martínez Paula, Arpita Saha, María A. Blasco

**Affiliations:** 1 Telomeres and Telomerase Group, Molecular Oncology Program, Spanish National Cancer Centre (CNIO), Madrid 28029, Spain

**Keywords:** telomeres, telomerase

## Abstract

Telomerase is a cellular enzyme that adds telomeric DNA sequences to the ends of chromosomes, aiding in preserving genome stability. The enzyme is formed by a protein with reverse transcriptase activity (TERT) and an RNA molecule that provides the sequence used as a template (TERC). These two components must interact precisely for telomerase to function, and their compatibility varies across species. Understanding whether the human protein component can function with the RNA molecules of other species is important for the development of therapies that activate telomerase, particularly those aimed at treating disorders associated with short telomeres. Here, we investigate the ability of the human TERT to interact with the telomerase RNA from several mammalian species commonly used in preclinical research, including monkey, pig, dog, rabbit, rat, and mouse. We introduced the human TERT into primary lung fibroblasts from each species and assessed its capacity to generate telomeric repeats in cell extracts. We also analyzed telomere length during extended cell culture to determine whether telomeres lengthened over time. Human TERT formed an active complex with the telomerase RNA from monkey, pig, rabbit, and rat *in vitro*, but not with that from dog or mouse. However, only monkey and human cells showed progressive telomere lengthening during long-term culture. These results reveal that only non-human primate cells support full functional activity of the human telomerase protein in a cellular context, underscoring their suitability as preclinical models for telomerase-based therapeutic strategies.

## INTRODUCTION

Linear chromosomes terminate in specialized chromatin domains, termed telomeres, which preserve chromosome integrity by preventing aberrant DNA degradation and inappropriate activation of DNA repair pathways; therefore, they are essential for ensuring chromosome stability [1, 2]. In mammals, telomeres consist of arrays of TTAGGG repeats whose overall length varies substantially between species, and the rate of telomere shortening in each species has been correlated to species longevity [3]. Because conventional DNA polymerases are unable to fully replicate chromosome termini, telomeric DNA progressively erodes with successive rounds of cell division, the so-called “end-replication problem” [4, 5]. Telomere shortening to a critically short length is proposed to be a primary molecular cause of senescence and age-associated diseases [6]. Telomerase is a ribonucleoprotein enzyme whose catalytic activity resides in the telomerase reverse transcriptase subunit (TERT) and whose template for DNA synthesis is provided by an associated RNA molecule (TERC), thus compensating for the telomere erosion caused by cell divisions [7]. Telomeric DNA is bound by the shelterin complex, a multiprotein assembly that plays essential roles in chromosome end protection and regulation of telomerase access [8]. The shelterin complex is composed of six core proteins: the telomeric repeat binding factor 1 and 2 (TRF1 and TRF2), the TRF1-interacting protein 2 (TIN2), protection of telomeres protein 1 (POT1), TIN2-interacting protein (TPP1), and the repressor/activator protein 1 (RAP1) [1, 2, 9]. TPP1 recruits POT1 to telomeres [10]. In addition, TPP1 is required for the recruitment of telomerase to chromosome ends *in vivo* [11, 12].

Several human disorders are caused by germline mutations in genes encoding proteins involved in telomere maintenance and repair, collectively known as telomere biology diseases (TBDs), telomeropathies or telomere syndromes [13, 14]. Impaired telomere maintenance leads to a premature decline in the regenerative capacity of tissues, ultimately resulting in the onset of disease. In humans, telomere biology diseases are most commonly linked to Hoyeraal–Hreidarsson syndrome, dyskeratosis congenita, aplastic anemia, and pulmonary and liver disorders, and are currently regarded as manifestations of a disease spectrum rather than as separate clinical entities [13–16]. Despite their broad and complex clinical heterogeneity, all of these disease conditions share the presence of critically short telomeres. Both the age of onset and the severity of clinical symptoms vary considerably among affected individuals. A hallmark of these syndromes is the early loss of tissue regenerative potential, impacting tissues with both high and low proliferative capacity [13, 15].

Therapeutic strategies based on telomerase activation are being developed to treat and prevent telomere-associated diseases, namely aging-related diseases and telomere biology diseases. Previous work from our group has explored the use of adeno-associated viral vectors carrying the homologous mouse *Tert* to transiently induce telomerase activity in mouse adult tissues, resulting in telomere elongation, improved tissue function, delayed onset of age-associated pathologies, and increased longevity [17–19]. *TERT* gene therapy represents a promising therapeutic strategy for human telomere biology diseases associated with telomerase mutations and critically short telomeres. Using a preclinical mouse model of aplastic anemia driven by telomere shortening in the bone marrow (BM), we previously showed that AAV9-*Tert* treatment effectively rescued both aplastic anemia and overall survival by promoting telomere elongation in peripheral blood and BM cells [20]. In line with these findings, AAV9-*Tert* therapy was also demonstrated to be beneficial in a mouse model of pulmonary fibrosis caused by short telomeres, where it restored telomere length in alveolar type II cells and increased their proliferative capacity [19, 20]. To further develop this therapy from preclinical to clinical stage, it is necessary to study the ability of the human telomerase catalytic subunit to lengthen telomeres in other animal species than mouse, in which human TERT is not functional [21–23]. In this work, we have tested the cross-species compatibility of human TERT in mouse, rat, rabbit, canine, porcine, and Cynomolgus monkey lung fibroblasts.

## RESULTS

### Heterologous expression of human TERT in primary lung fibroblasts of various mammalian species

To address whether human telomerase (hTERT) is functionally active in other mammalian species, the human hTERT gene was cloned into the lentiviral vector pLOC, where the expression of hTERT is driven by the human cytomegalovirus promoter (hCMV). Lentiviral particles were produced by transfecting HEK293T cells with either the empty pLOC or with PLOC-hTERT together with the packaging and envelope expressing plasmids pMDLg/pRRE, pRSV-Rev and pCMV-VSV-G. Culture supernatant containing the viral particles was used to transduce primary lung fibroblasts from rat, rabbit, canine, porcine, and Cynomolgus monkey. Primary lung human fibroblasts and primary BJ fibroblasts were also transduced as positive controls. Infected cells were selected on Blasticidin-containing media. Cells were then successively passaged to allow for telomere elongation associated to cell division and samples were taken 10, 40 and 80 days post-infection for further analysis (see experimental setup in Figure 1).

**Figure 1:**
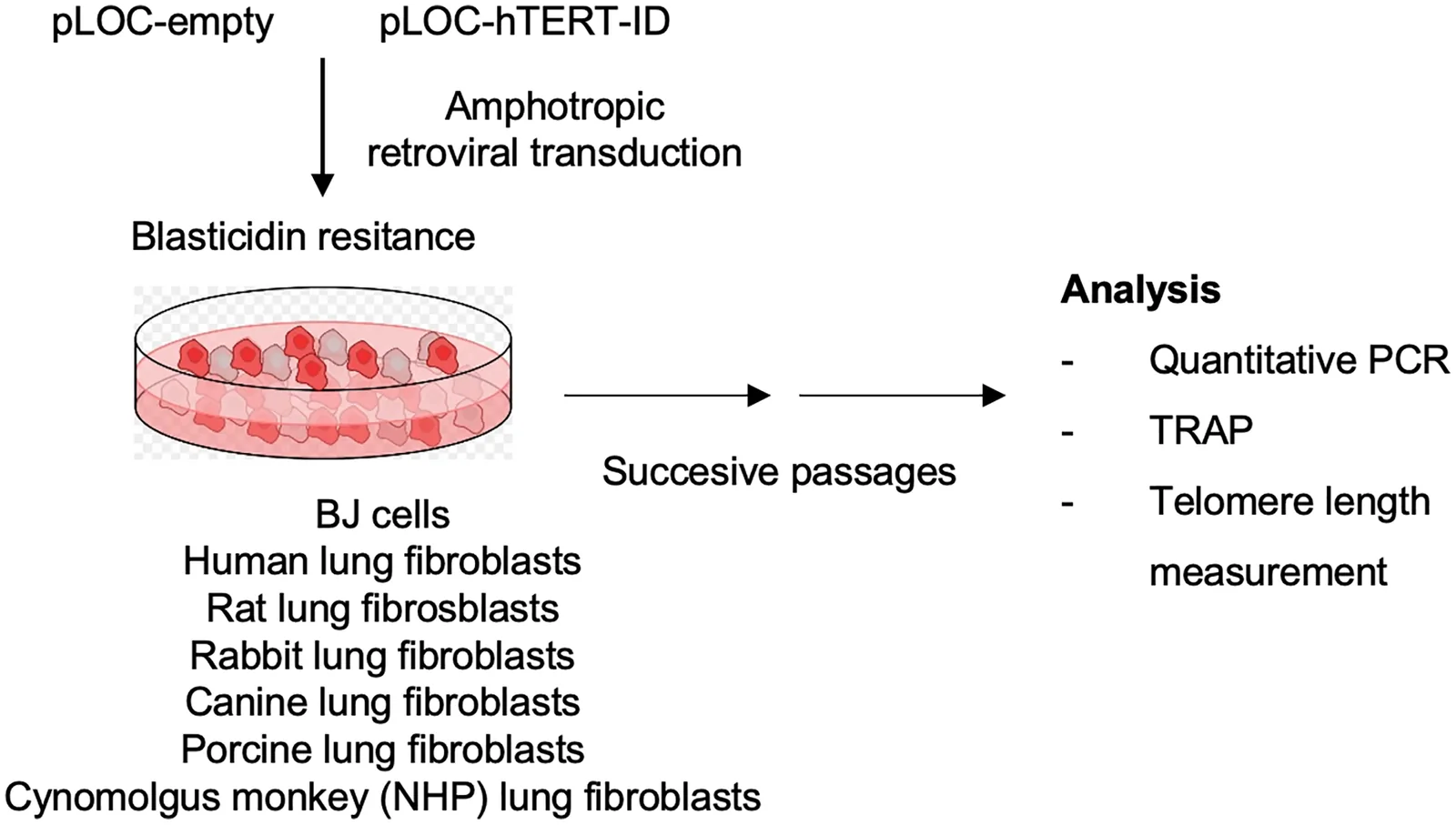
**Experimental layout.** Lung fibroblasts from rat, rabbit, dog, pig and Cynomolgus monkey were transduced with either pLOC-empty or with pLOC-hTERT. Primary lung human fibroblasts and BJ fibroblasts were also transduced as positive controls. Infected cells were selected on Blasticidin-containing media. Cells were then successively passaged, and samples were taken 10, 40, and 80 days post-infection for further analysis.

Transcriptional expression of hTERT was analyzed at 10 days post-infection by RT-q-PCR. All the species infected with pLOC-hTERT showed expression of hTERT mRNA while no PCR amplification was detected in control pLOC-empty infected cells (Figure 2).

**Figure 2:**
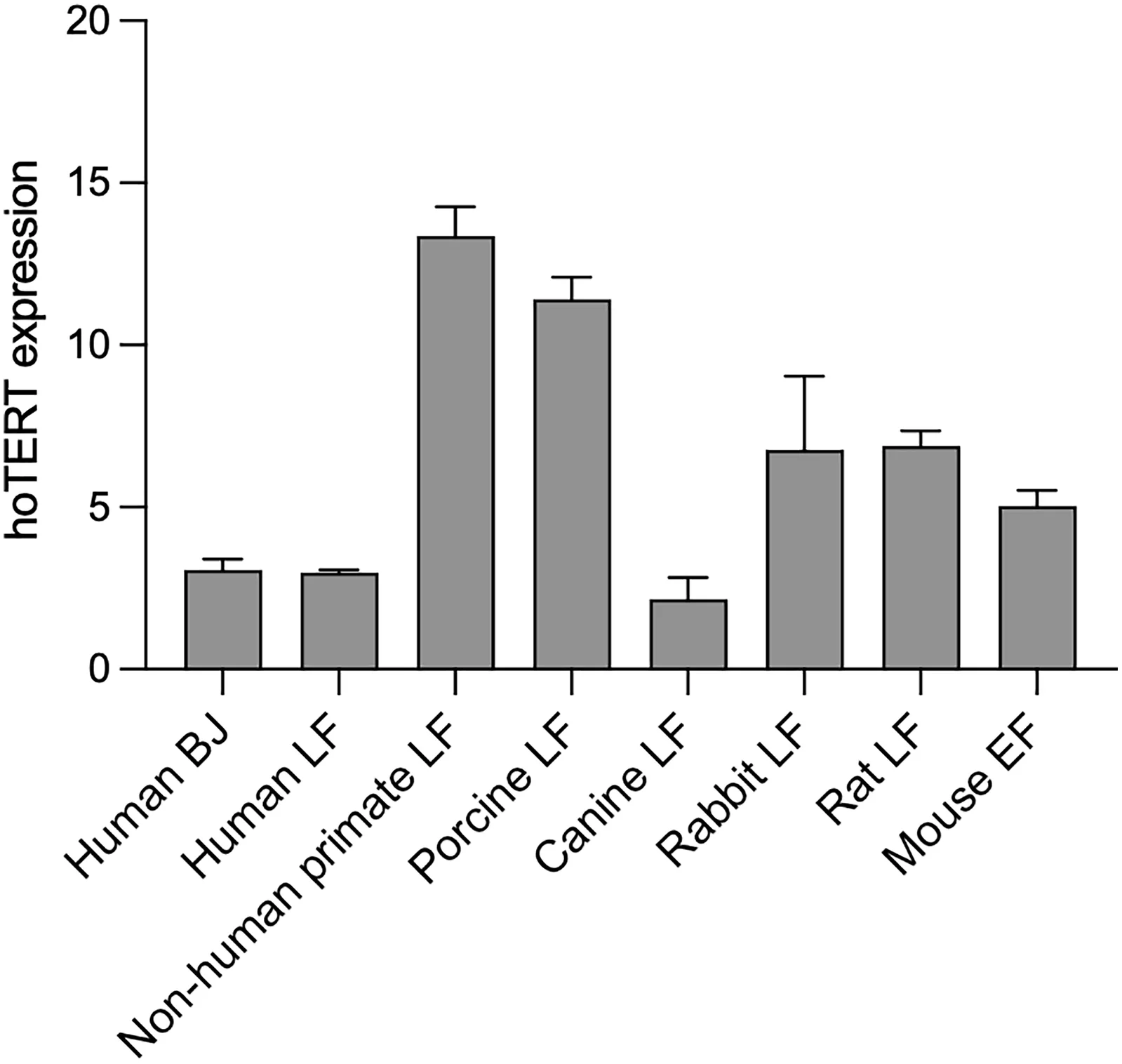
**hTERT is expressed in different species.*** hTERT* expression levels in pLOC-hTERT infected human BJ, human lung fibroblasts, non-human primate lung fibroblasts, porcine lung fibroblasts, canine lung fibroblasts, rabbit lung fibroblasts, rat lung fibroblasts, and in *Tert^−/−^* mouse embryonic fibroblasts. pLOC-empty infected cells were used as negative controls and hTERT expression was undetected in all cell lines. Transcriptional *hTERT* was analyzed 10 days post-infection, and samples were analyzed in triplicate. The y-axis represents the mean 2ΔCT, and the error bars represent the standard error.

### *In vitro* cross-species activity of hTERT

Telomerase is composed of a reverse transcriptase catalytic subunit (TERT) and an RNA template (Terc) that associate and function in a co-dependent manner to synthesize telomeric repeats at the 3’ end of the G-strand overhang and elongate the telomere [24]. To address whether the human catalytic subunit of telomerase (TERT) can form active complexes with the RNA component of mouse, rat, rabbit, canine, porcine and Cynomolgus monkey, we performed Telomerase Repeat Addition Protocol (TRAP) in cells transduced either with pLOC-empty or pLOC-hTERT (Figure 3). The TRAP results show that human hTERT was able to generate TTAGGG repeats *in vitro* using the telomerase RNA components from monkey, pig, rabbit and rat (Figure 3). In contrast, human hTERT did not show any *in vitro* activity in the case of dog and mouse cells. As positive controls, we transduced TERT into telomerase-negative human lung fibroblasts as well as BJ fibroblasts, and in all cases transduced hTERT reconstituted telomerase activity (Figure 3).

**Figure 3:**
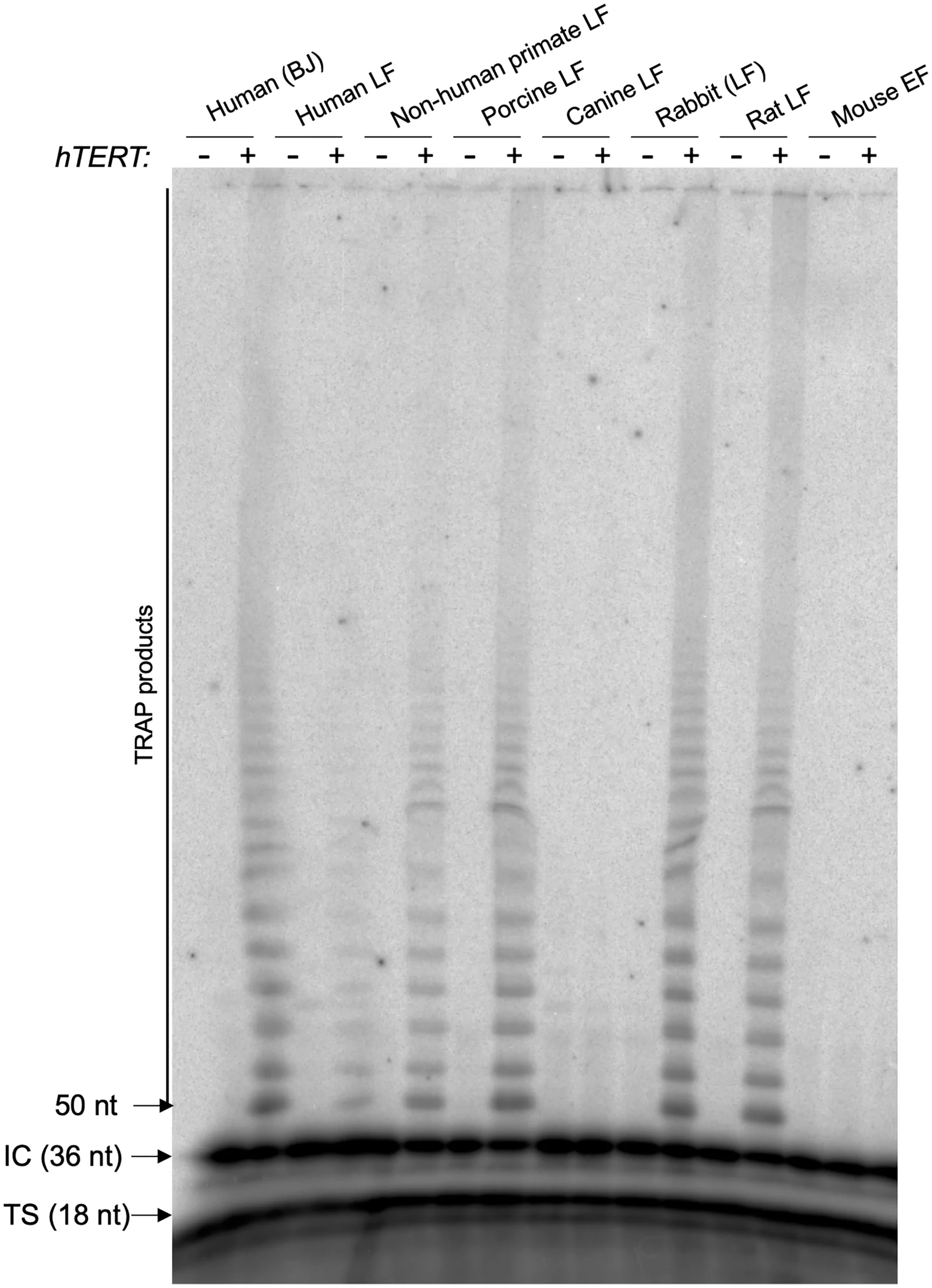
**hTERT is active by TRAP in human, non-human primate, porcine, rabbit, and rat cells, and inactive in canine and mouse cells.** Telomerase Repeat Addition Protocol (TRAP) in cells transduced either with pLOC-empty or pLOC-hTERT. Abbreviations: IC: internal control for the PCR efficiency; TS: telomerase substrate. The number of nucleotides (nt) of IC, TS and the first TRAP amplified fragment are indicated.

### *In vivo* cross-species activity of hTERT

Transduced cells with either the control vector or with the vector containing hTERT were maintained in culture through successive passages to allow for potential telomere elongation by telomerase associated with cell division, as telomerase is active during late S Phase [24]. To measure telomere length, cells were plated in Opera plates at 10, 40, or 80 days post-infection (dpi), and we performed quantitative high throughput-telomere quantitative FISH (HT-QFISH) to analyze telomere fluorescence intensity as a readout of telomere length [25]. At 10 dpi, no telomere intensity changes were observed in any of the species under study (Figure 4A and Supplementary Figure 1A), indicative of a lack of telomere elongation at early passages. However, at 40 dpi and 80 dpi, a significant increase in telomere fluorescence intensity was detected in human and in monkey cells, indicating progressive telomere lengthening owing to reconstitution of a productive telomerase enzyme able to extend telomeres. In contrast, increasing passages of rabbit, rat, and porcine cells showed a significant decrease in telomere fluorescence intensity, indicating a lack of an active telomerase complex on telomeres. Both canine and mouse cells transduced with hTERT did not survive after 10 dpi (Figure 4B and Supplementary Figure 1B), indicating the toxicity of the human TERT. Similarly, heterologous expression of hTERT in mouse cells leads to cell senescence characterized by the induction of the senescent markers p19ARF, p16INK4 and p21 (Figure 5).

**Figure 4:**
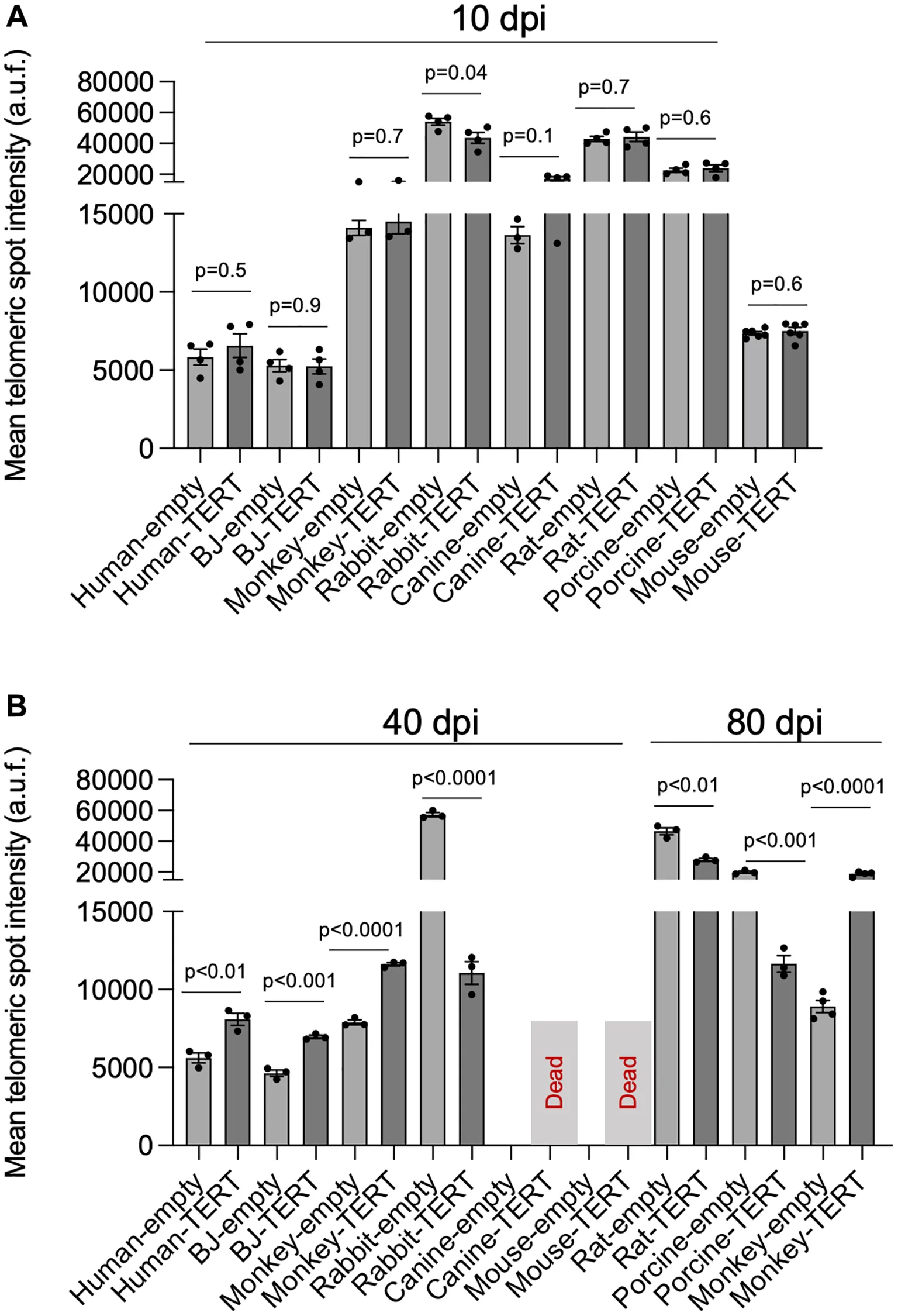
**hTERT progressively lengthens telomeres with passages in human and in non-human primate cells.** (**A**, **B**) Telomere length analysis by HT-qFISH in human BJ, human lung fibroblasts, non-human primate lung fibroblasts, porcine lung fibroblasts, canine lung fibroblasts, rabbit lung fibroblasts, rat lung fibroblasts, and in *Tert^−/−^* mouse embryonic fibroblasts transduced either with pLOC-empty or pLOC-hTERT. Cells were analyzed at 10 (**A**), 40, or 80 (**B**) days post-infection (dpi). The assays were performed in 3–4 replicas. The bars represent the mean value, and the error bars the standard error. The statistical significance was calculated by an unpaired Student’s *t*-test (two-tailed).

**Figure 5:**
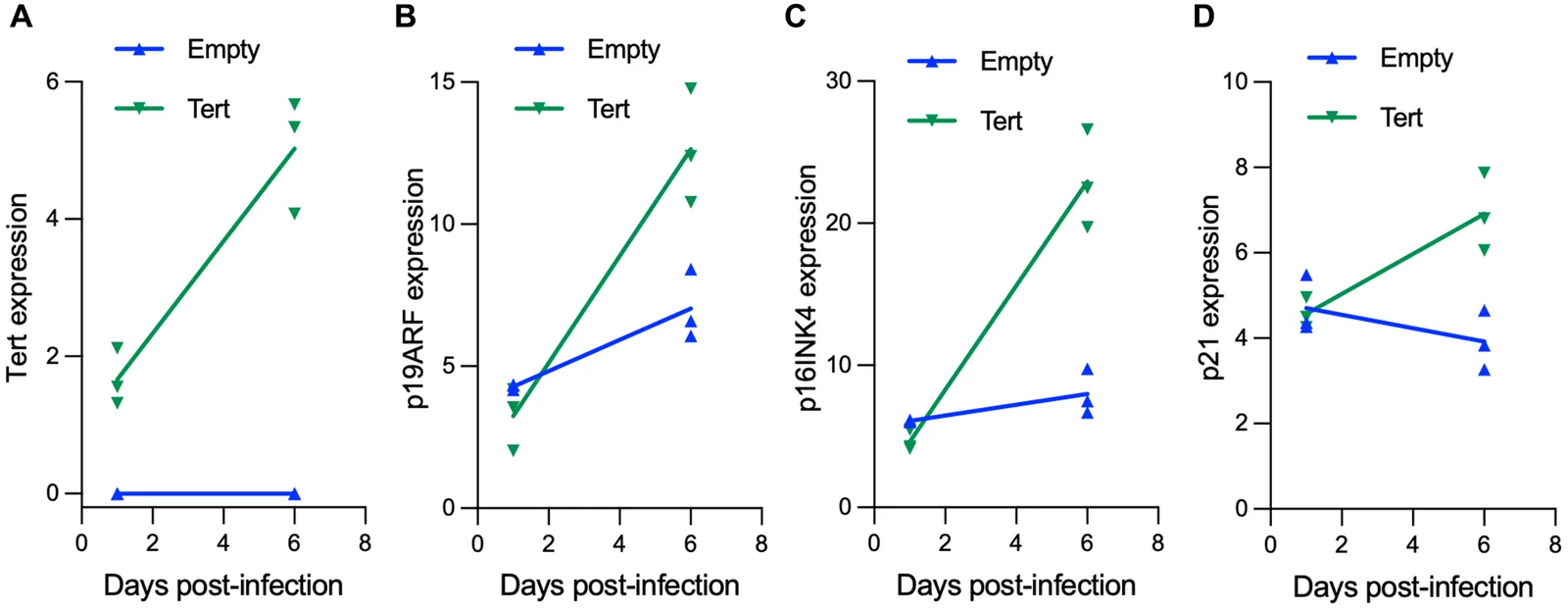
**hTERT expression in mouse fibroblasts induces cell senescence.** Mouse embryonic fibroblasts were transduced either with pLOC-empty or pLOC-hTERT, and samples were taken at day 1 and at day 6 post-infection. (**A**–**D**) hTERT (**A**) and the senescence markers p19ARF (*Cdkn2A* gene) (**B**), p16Ink4A (*Cdkn2A* gene) (**C**) and p21 (*Cdkn1A* gene) (**D**) transcriptional expression was analyzed by RT-q-PCR.

## DISCUSSION

Telomerase functions as a ribonucleoprotein enzyme whose activity depends on the coordinated interaction between the telomerase reverse transcriptase subunit (TERT) and its associated RNA component (Terc) [26, 27]. Despite the conservation of overall structural features among vertebrate telomerase RNA molecules, their primary sequences display substantial divergence across species, which can limit functional interchangeability [21, 28]. Consistent with earlier studies, our data confirm that the human TERT protein is unable to generate an active telomerase complex when paired with the mouse telomerase RNA component *in vitro* [23]. However, human telomerase RNA (hTR) forms a functional telomerase complex with mouse TERT *in vitro* and *in vivo* [23, 29]. Previous reports have shown that heterologous expression of hTERT in mouse cells interferes with endogenous telomerase function and can trigger loss of cellular viability [22]. In this work, we show that heterologous expression of hTERT in primary mouse embryonic fibroblasts (MEF) that are *knock-out* for mouse *mTert* is toxic, in agreement with previous reports [22]. Human TERT was previously shown to be functionally compatible with rabbit telomerase RNA (rTR) in an *in vitro* TRAP assay, although the consequences for telomere maintenance in living cells remained unexplored [30, 31].

Here, we set out to explore the cross-species activity of human TERT. It is important for the preclinical and clinical development of potential human telomerase activation strategies for the treatment of telomere syndromes and age-related diseases. So far, preclinical studies have been carried out in mouse using mTERT and have shown promising results [18–20]. Since hTERT is not functional in mice and its ectopic expression results in cell senescence (this work and [22]), other animal model than mouse must be identified to address the efficacy and potential toxicity effects of heterologous expression of human TERT. A prerequisite for human TERT to be functionally active to elongate telomeres in other organisms is that the catalytic subunit (hTERT) is compatible with the homologous telomerase RNA component of the specie under study. In this work, we show that the human telomerase catalytic subunit can assemble into biochemically active complexes with telomerase RNA components from several mammalian species, including Cynomolgus monkey, pig, rabbit, and rat, while it does not with dog and mouse in an *in vitro* TRAP assay. The absence of TRAP activity in dog and mouse cells could either be due to the inability of hTERT to recognize/bind to their endogenous telomerase RNA component or to that the assembled ribonucleoprotein complexes are inactive. Importantly, our telomere length analyses across serial passages reveal that biochemical compatibility *in vitro* does not necessarily translate into functional telomere elongation within a cellular context in either rabbit, rat, or pig. Indeed, in these species, telomeres became progressively shorter with increasing passage number compared to control samples transduced with the control vector. The shorter telomeres observed in hTERT transduced rabbit, rat and pig cells as compared to control counterparts could be explained by interference of the heterologous telomerase with the endogenous one resulting in its inactivation. In contrast, hTERT was fully capable of progressively lengthening the telomeres with increasing passages in human as well as in Cynomolgus monkey cells. These findings likely reflect the closer evolutionary relationship between humans and non-human primates, which may preserve species-specific requirements for telomerase regulation at chromosome ends.

The assembly, regulation, and telomere recruitment of telomerase *in vivo* involve multiple coordinated steps and depend on a network of accessory factors that extend beyond the catalytic core [32–34]. For instance, human POT1 functions both as a positive and a negative regulator of telomerase since POT1 and its interacting partner TPP1 have been shown to have a role in recruiting telomerase to telomeres as well as to inhibit telomerase access to telomeres [32, 33]. Lack of telomere lengthening *in vivo* in those cases in which we observed *in vitro* telomerase activity by TRAP could also be explained by incompatibility with non-human shelterin components and/or defective nuclear translocation. Therefore, assays of telomerase catalytic activity *in vitro* do not reflect the physiological requirements that recruit the telomerase complex to telomeres and those that regulate its activity and processivity. In this work, by analyzing not only the *in vitro* activity of hTERT with other species telomerase RNA components but also the *in vivo* activity of these cross-species telomerase complexes in lengthening telomeres, we identified non-human primates as the most suitable preclinical models for evaluating telomerase-based therapeutic approaches that rely on human TERT activity.

## MATERIALS AND METHODS

### Cell lines

Human primary lung fibroblasts (H-6013, Innoprot), Porcine primary lung fibroblasts (P-6013, Innoprot), Canine primary lung fibroblasts (D-6013, Innoprot), Rabbit primary lung fibroblasts (N-6013, Innoprot), Rat primary lung fibroblasts (RA-6013, Innoprot) and Cynomolgus Monkey primary lung fibroblasts (NHP-PC119, Creative Biolabs), BJ fibroblasts (ATCC-CRL-2522) and telomerase-deficient *Tert^−/−^* Mouse embryonic fibroblasts.

### Retroviral transduction

The human telomerase (*hTERT*) was cloned into the lentiviral vector pLOC (Horizon, #OHS5832). The empty vector pLOC Horizon or pLOC-hTERT together with pMDLg/pRRE (Addgene #12251), pRSV-Rev (Addgene #12253), and pCMV-VSV-G (Addgene #8454) vectors were transfected into HEK293T cells for amphotrophic lentiviral particle production. The different cells lines were transduced with either the empty or the telomerase-containing viral particles and selected for Blasticidin resistance (2–10 mg/ml). Cells were successively passaged, and samples were taken at 10, 40, and 80 days post-infection.

### Telomerase and senescence marker expression

Total RNA from cells was extracted with the RNeasy kit (74106, QIAGEN) and reverse transcribed using the iSCRIPT cDNA synthesis kit (1708891, BIO-RAD) according to the manufacturer’s protocol. Quantitative real-time PCR was performed with the QuantStudio 6 Flex (Applied Biosystems, Life Technologies) using Go-Taq Green Master Mix (M7123, Promega) according to the manufacturer’s protocol. All values were obtained in triplicates. We determined the relative expression in each sample by calculating the 2ΔCT value. For each sample, 2ΔCT was normalized to control 2ΔCT mean. The primers used for *hTERT* are hTert-F (5′-TATGTTGTAGGCGCTCGGAC-3′) and hTert-R (5′-CTCCACGCCCGGTGTATATC-3′), for p19Arf (*Cdkn2A*) are p19-F (5′-AATCTGCACCGTAGTTGAGCA-3′) and p19-R (5′-GGGTCGCAGGTTCTTGGTC-3′), for p16Ink4A (*Cdkn2A*) are Cdkn2A-F (5′-GGGTTTCGCCCAACGCCCCGA-3′) and Cdkn2A-R (5′-TGCAGCACCACCAGCGTGTCC-3′), and for p21 (*Cdkn1A*) are Cdkn1A-F (5′-TCCCGACTCTTGACATTGCT-3′) and Cdkn1A-R (TCCCGACTCTTGACATTGCT-3′). The Actin gene (*Actb*) was used as a housekeeping gene using the primers Actin-F (5′-GGCACCACACCTTCTACAATG-3′) and Actin-R (5′-GTGGTGGTGAAGCTGTAG-3′). Similar primers were used in all the species under study since both primers anneal in the *Actb* gene of all the species.

### Telomeric repeats amplification protocol (TRAP)

Telomerase activity was measured by TRAP, as previously described [35]. Briefly, cells were lysed with CHAPS buffer, and the soluble fractions were freshly frozen in liquid nitrogen and stored at −80 °C. Cell extracts (1 μg) were incubated with telomeric primers for a 60 min initial extension step at 30°C. The extended reaction was subjected to PCR amplification (25 cycles of 30 s at 94°C, 30 s at 59°C, 30 s at 72°C) in the presence of ^32^P end-labeled telomeric primer. The PCR reactions were resolved in 8% polyacrylamide, 7 M urea gel electrophoresis in 0.5X TBE buffer. The gel was dried, exposed to a phosphor-imager, and scanned by a Typhoon scanner.

### High-throughput quantitative fluorescence *in situ* hybridization (HT-Q-FISH) analysis

Cells were plated in a 96-well plate coated with poly-lysine (Greiner Bio-One, Inc.; catalog no. 655087) and fixed in Methanol: Acetic acid (3:1) for 15 minutes. Plates were stored at −20 °C with the fixative solution. For Q-FISH hybridization, plates were dried at 37ºC overnight, and cells were then rehydrated with 1xPBS and fixed with 4% formaldehyde, followed by digestion with pepsin/HCl and a second fixation with 4% formaldehyde. Samples were dehydrated with increasing concentrations of EtOH (70%, 90%, 100%) and incubated with the telomeric (TTAGGG) probe labelled with Cy3 at 85°C for 3 min, followed by 1 h at room temperature in a wet chamber. Samples were extensively washed with 50% formamide and 0.08% TBS-Tween 20, followed by TBST containing 1 μg/mL DAPI (4′,6-diamidino-2-phenylindole, dihydrochloride; Life Technologies; catalog no. D-1306) to stain the nuclei [25]. Next, the plate was washed 1 × 5 min with a plate shaker. Confocal images were captured using the Opera Phenix High-Content Screening System (PerkinElmer). Images were analyzed with Harmony High-Content Analysis Software (PerkinElmer).

### Statistical methods

The statistical significance was calculated by an unpaired Student’s *t*-test (two-tailed).

## Supplementary Materials

Supplementary Figure 1
