# Supplementary material for: Cross species activity of TERT human telomerase component
Source: Aging (Albany NY). 2026 Apr 13;18(1):369–79. doi: 10.18632/aging.206372 (PMC13632861; doi:10.18632/aging.206372)
Supplement: Supplementary file 1 [file aging-18-1-206372-s001.pdf]

SUPPLEMENTARY FIGURE

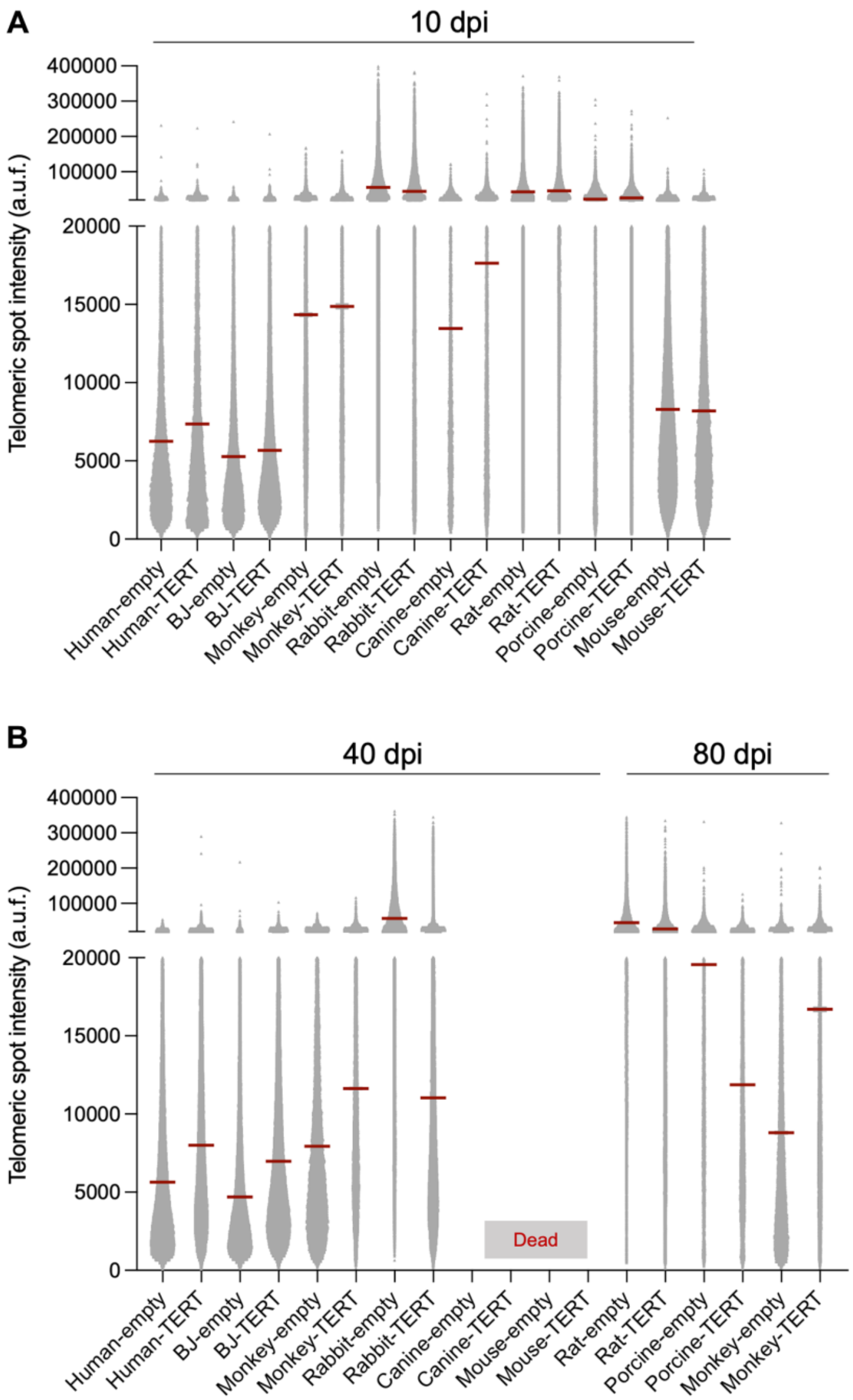

Supplementary Figure 1. Telomere distribution at different cell passages. Distribution of single telomere focus intensity in human BJ, human lung fibroblasts, non-human primate lung fibroblasts, porcine lung fibroblasts, canine lung fibroblasts, rabbit lung fibroblasts, rat lung fibroblasts, and in mouse embryonic fibroblasts transduced either with pLOC-empty or pLOC-hTERT. Cells were analyzed at 10 (**A**), 40, or 80 (**B**) days post-infection (dpi). The assays were performed in 3-4 replicas, and the individual telomere focus intensity was pooled.
